# Three-dimensional co-culturing reveals human stem cell-derived somatostatin interneurons with subclass expression

**DOI:** 10.1016/j.stemcr.2025.102634

**Published:** 2025-09-09

**Authors:** Andreas Bruzelius, Christina-Anastasia Stamouli, Anna-Lena Hölldobler, Constanza Aretio-Medina, Efrain Cepeda-Prado, Edoardo Sozzi, Germán Ramos Passarello, Gianluigi Nocera, Jessica Giacomoni, Victor Olariu, Daniella Rylander Ottosson

**Affiliations:** 1 Regenerative Neurophysiology, Lund Stem Cell Centre, MultiPark Strategic Area in Neuroscience, Department of Experimental Medical Science, Faculty of Medicine, Lund University, 221 84 Lund, Sweden; 2 Developmental and Regenerative Neurobiology, Lund Stem Cell Centre, MultiPark Strategic Area in Neuroscience, Department of Experimental Medical Science, Faculty of Medicine, Lund University, 221 84 Lund, Sweden; 3 Computational Science for Health and Environment, Centre for Environmental and Climate Science, Faculty of Science, Lund University, 223 62 Lund, Sweden

**Keywords:** somatostatin, MGE, GABAergic interneurons, astrocytes, glia precursor, hESC, 3D cell culture, neuroscience, disease modelling, patch-clamp electrophysiology

## Abstract

Cortical interneuron deficiencies, particularly involving the somatostatin (SST) subtypes, contribute to neurological and neuropsychiatric disorders. These interneurons are difficult to derive *in vitro* from human embryonic stem cells (hESCs) due to their late embryonic development and dependence on glial interaction. To this end, we developed a three-dimensional co-culture model of hESC-derived neurons, enabling long-term development, functional maturity, and neuron-glial interaction. Under these conditions, hESCs successfully differentiated into functional GABAergic interneurons expressing the SST gene and protein within 50 days. Single-nuclei RNA sequencing revealed transcripts for SST subclasses, including Martinotti, non-Martinotti, and long-projecting neurons, that have not yet been described for hESC cultures. Upon injection into forebrain organoids, the interneuron progenitors spread and functionally matured while retaining their SST subclass identities, suggesting cell-intrinsic fate specification. Our *in vitro* model provides a robust platform for studying human SST interneurons, offering new avenues for investigating their role in health and disease.

## Highlights

• Successful SST interneuron generation: 3D model yields SST neurons from hESCs in 50 days
• Subtype discovery: identified diverse SST interneurons from hESCs via RNA sequencing
• Organoid integration: injected cells matured and retained SST subtype identity
• Research platform: provides a new model to study SST in brain health and disease

## Teaser

Rylander and colleagues present a 3D co-culture model for successful generation and maturation of somatostatin interneurons from human embryonic stem cells. This system reveals previously uncharacterized SST subtypes and supports integration into forebrain organoids, offering a powerful platform to investigate interneuron development and their roles in neurological and psychiatric disorders.

## Introduction

Neuronal cells of the neocortex can be broadly classified into two main groups: excitatory pyramidal cells (70%–80%) and inhibitory GABAergic interneurons (20%–30%). Although fewer in number, GABAergic interneurons are crucial for cortical circuitry development (Ferrer and De Marco García, 2022) and sensory processing (Tremblay et al., 2016). During development, these interneurons migrate from the medial and caudal ganglionic eminences (MGE and CGE, respectively), located in the ventral forebrain (FB), to the dorsal FB. There, they mature during late gestational to early postnatal periods, acquiring unique region-specific subtype identities. The main interneuron populations in the cortex are the somatostatin (SST) and parvalbumin (PV) subtypes, both originating in the MGE (Markram et al., 2004; Tasic et al., 2016, 2018; Wu et al., 2023). Notably, the SST interneurons represent the most diverse GABAergic subtype to date, with over 13 subclasses with distinctive morphoelectrical and transcriptomic profiles being identified in the adult mouse cortex using Patchseq, including Martinotti, non-Martinotti, and long-range projecting neurons (Fisher et al., 2024; Gouwens et al., 2020; Wu et al., 2023).

Cortical SST interneurons regulate excitatory input across the cortex. These cells gate the flow of information in the cerebral cortex and play a pivotal role in cortical slow-wave generation. This function makes SST interneurons incredibly important for physiological brain function and emotion (Jackson et al., 2024). Dysfunction in SST interneurons has been linked to neurological and neuropsychiatric disorders, often characterized by reduced SST expression (Song et al., 2021; Bershteyn et al., 2023). Moreover, large-scale clinical genetic studies demonstrated that gene mutations affecting the development and function of MGE-derived interneurons are associated with disorders such as epilepsy and schizophrenia (Batiuk et al., 2022). This underscores the importance of studying human-derived interneurons for disease modeling and the development of restorative cell therapies.

Although protocols for generating MGE progenitors and GABAergic neurons from pluripotent stem cells have been previously established (Close et al., 2017; Liu et al., 2013; Maroof et al., 2013; Ni et al., 2019; Nicholas et al., 2013), these cell preparations demonstrate a protracted maturation of more than 200 days, often yielding heterogeneous populations, including oligodendrocytes, astrocytes, and non-cortical interneurons, such as cholinergic or GABAergic striatal interneurons (Nicholas et al., 2013). This is different from *in vivo* transplantation in the rodent brain, where human MGE can differentiate into subtype-specific interneurons in a shorter time (Upadhya et al., 2019). As a result, current *in vitro* models of human subtype-specific SST interneurons remain limited, requiring extensive developmental timelines that are often incompatible with standard cell culture conditions and cell viability (Bershteyn et al., 2017; Close et al., 2017).

Herein, we developed a human three-dimensional (3D) co-culture system that allows for easy long-term maintenance *in vitro* while facilitating synaptic complexity. Using a modified high-purity MGE progenitor differentiation protocol from human embryonic stem cells (hESCs), we matured the cells in self-formed spheroids that were co-cultured with glial precursor cells or astrocytes to support functional maturation and network connectivity. We found that hESCs successfully differentiated into GABAergic pallial interneurons and showed functional neuronal properties with GABAergic network connectivity enhanced by astrocyte co-culture. Importantly, the 3D culturing system promoted differentiation into SST interneurons within 50 days *in vitro* (DIV). These interneurons showed SST subclass identities, including long-projecting neurons and SST-TH subclass. The MGE-like progenitors were able to survive, integrate, and functionally mature in human brain organoids upon injection while maintaining their phenotypic SST fate. These results highlight the potential of a 3D co-culture model as a valuable tool for disease modeling in interneuropathies linked to the SST subtype.

## Results

### Differentiation of hESCs into MGE progenitors and 3D spheroid formation

**Figure 1:**
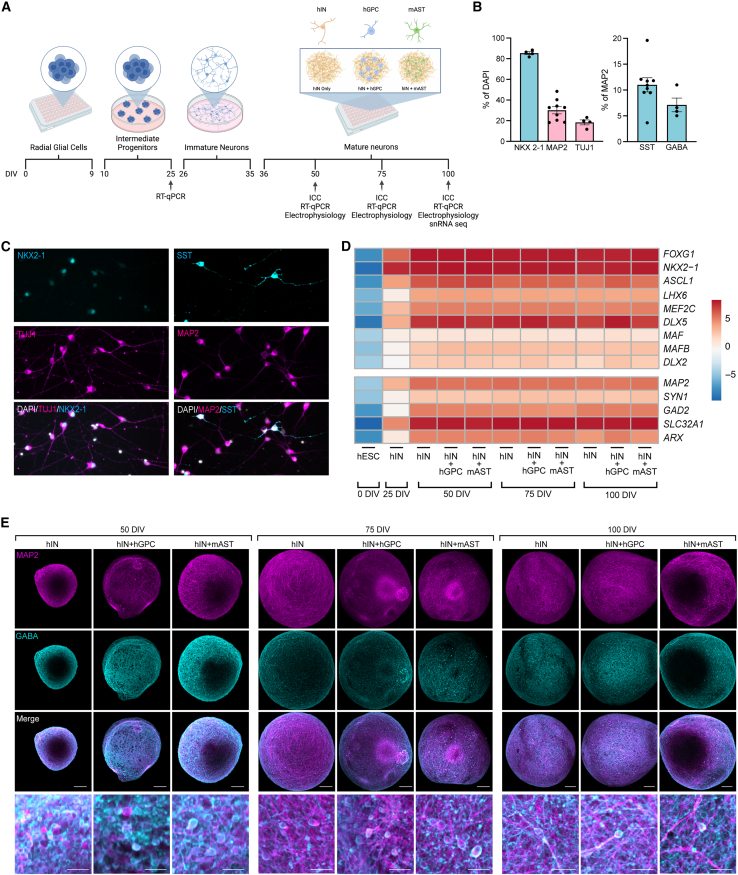
hESCs differentiate into GABAergic interneurons in 3D co-cultures for up to 100 DIV (A) Schematic representation of MGE-like cell induction protocol and spheroid formation. (B) Quantification of cells expressing NKX2.1 (*n* = 4), MAP2 (*n* = 7), and TUJ1 (*n* = 4) as well as SST (*n* = 7) and GABA (*n* = 4) in MAP2^+^ cells at 35 DIV (*n* = independent replicate, all values are represented as mean ± SEM). (C) Immunofluorescence showing expression of NKX2.1, TUJ1, MAP2, and SST at 35 DIV. (D) Heatmap showing relative gene expression levels of different time points across the differentiation protocol (see also Figure S1A; Table S1 for statistics). (E) Maximum intensity projection confocal images showing MAP2 and GABA expression throughout the entire spheroid across time points and culture conditions (see also Figure S1B). Scale bars for (E): 100 μm; zoom = 10 μm; hIN, human interneurons; hGPC, human glial progenitor cells; mAST, mouse astrocytes; DIV, days *in vitro*.

MGE-like progenitors were derived using a previously published protocol with slight modification (Nicholas et al., 2013). After initial patterning, cells were replated as a single-cell suspension in a monolayer culture, and at 35 DIV, initial immunofluorescence was done to confirm correct patterning (Figure 1C). This showed 88.3% ± 1.4% of the cells positive for the MGE-specific progenitor marker NKX2.1 (Figure 1B), indicating the induction of the MGE GABAergic neuronal fate. Moreover, 30.1% ± 3.5% and 18.3% ± 2.4% of the cells were positive for the neuronal markers microtubule-associated protein 2 (MAP2) and class III beta-tubulin (Tuj1), respectively (Figure 1B). With mature neurons observed as early as 35 DIV, we further investigated subtype-specific MGE markers, identifying 11.0% ± 1.4% co-expression of MAP2 and SST and 7.1% ± 1.3% co-expression of MAP2 and GABA (Figure 1B). After this initial GABAergic interneuron (IN) profile, cells were replated in ultra-low attachment plates to form 3D spheroids (Figure 1A) in combination with either human glial progenitor cells (hIN+hGPC), mouse astrocytes (hIN+mAST), or kept without co-culture as interneurons (hIN). Human glial progenitor cells (hGPCs) were uniquely available in our environment and differentiated from a long-term stem cell protocol (Giacomoni et al., 2020; Nolbrant et al., 2020). While mainly composed of PDGFRα^+^ cells, this co-culture provided both a human and glial-type comparison to the mouse astrocytic co-culture. Spheroid co-cultures were maintained in 3D for up to 50, 75, and 100 DIV for transcriptomic, histological, and functional assessment (Figure 1A).

### MGE-like progenitors can mature and be maintained for 100 DIV in 3D co-cultures

Gene expression levels were first analyzed with quantitative reverse-transcription PCR (RT-qPCR) and compared to the monolayer stage (25 DIV) and with the stem cell starting population (0 DIV, Figure 1D). This revealed early upregulation of the FB marker *FOXG1* and the MGE marker *NKX2-1* already at 25 DIV, which persisted throughout the spheroid maturation process, indicating a consistent ventral FB fate in 3D. Already after 50 DIV, there was a significant gene upregulation for MGE-associated transcription factors *ASCL1* and *LHX6* that was independent of co-culture condition (Tables S1C and S1D for statistics). Similarly, *MEF2C*, a marker linked to the maturation of GABAergic interneuron activity, was significantly upregulated in both co-culture conditions (Tables S1C and S1D; Figure 1D). *DLX5*, on the other hand, which promotes terminal differentiation of interneurons and subtype specification, showed higher upregulation only in the hIN+hGPC condition (Figure 1D), potentially due to GPC expression of this marker. A significant upregulation of *DLX2* was seen for all conditions, an upstream regulator of *DLX5*, driving subpallial GABAergic interneuron differentiation (Tables S1C and S1D) (Pla et al., 2018). While MGE-related markers showed consistent upregulation, there was no induction of *MAF* and only slight upregulation of *MAFB*, markers associated with pallidal interneuron fate (Sandberg et al., 2016; Vogt et al., 2014).

Mature neuronal genes were detected as early as 50 DIV, with significant upregulation of *MAP2* and an increase in *SYN1* expression (Tables S1C and S1D). Similarly, the mature GABAergic marker *GAD2* and *SLC32A1* encoding the vesicular GABA transporter (*VGAT*) were significantly upregulated at this time point. The interneuron migration marker *ARX* was upregulated in the hIN-only condition (Tables S1A and S1B for statistics). Glial markers were also seen in the 3D cultures, with a significant increase in *PDGFRA*, especially in the hIN+hGPC co-culture (Figure S1A). *GFAP* was upregulated in all culture conditions at later time points, including the hIN group, suggesting potential gliogenesis even without the addition of glial co-culture. Nevertheless, this upregulation reached significance only in the co-culture conditions (Tables S1C and S1D). Together, these data indicate successful differentiation into GABAergic neuronal fate in 3D cultures, particularly in the co-culture conditions (Figure 1D).

Transcriptional observations were further confirmed at the protein level across all time points (Figure 1E). Immunocytochemistry for MAP2 and GABA demonstrated widespread co-expression of these markers at 50 DIV, which was maintained until 100 DIV, confirming a stable neuronal GABAergic phenotype. The double-positive cells showed typical neuronal morphology and were distributed throughout the entire spheroid (Figure 1E). In line with the mRNA expression, few GFAP^+^ cells were detected in all three conditions, including in the hIN-only group from 75 DIV (Figure S1B). Expression of PDGFRα was, however, restricted to the hIN+hGPC group (Figure S1B). This represents an earlier time point than previously observed gliogenesis in conventional 2D monolayer cultures (Close et al., 2017) and suggests an accelerated developmental timeline in 3D.

Together, these results show successful differentiation into GABAergic interneurons in 3D co-cultures for up to 100 DIV.

To show the robustness of our protocol, we repeated the experiment for another hESC line (H9) in 3D with mAST co-culture. RT-qPCR data from this showed strong upregulation of *NKX2-1* and *FOXG1* already at 25 DIV of differentiation (Figure S1C). At 50 DIV in 3D spheroids, there was upregulation of important fate markers, i.e., *LHX6*, *DLX5*, *VGAT*, and *MAP2*, like RC17-derived spheroids (Figure S1C). At 35 DIV, immunofluorescence confirmed the correct patterning of interneurons, with expression of NKX2.1 (Figure S1D). We could also detect the expression of MAP2^+^ cells, some of which also co-expressed SST (Figure S1D), and immunocytochemistry at 50 DIV demonstrated the expression of MAP2 and GABA (Figure S1E), as well as expression of GFAP (Figure S1F), supporting the established 3D protocol for RC17-derived interneurons.

### Early astrocyte co-culture supports functional maturation and connectivity

**Figure 2:**
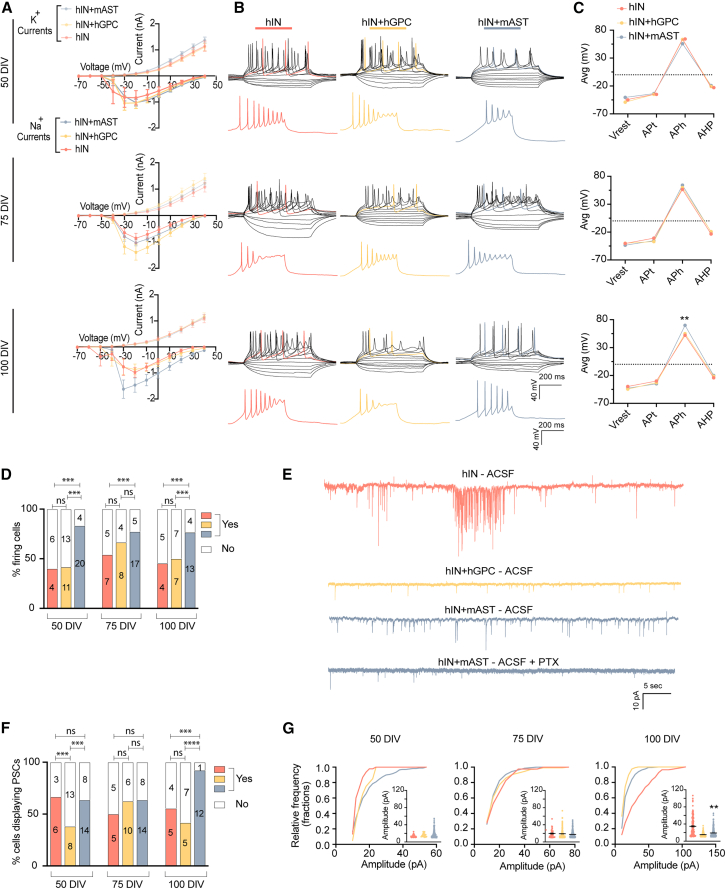
Early astrocyte co-culture supports functional maturation and connectivity (A) Inward Na^+^ and outward K^+^ currents plotted against voltage injection steps for all time points and conditions. All values are represented as mean ± SEM (50 DIV, hIN *n* = 11; hIN+hGPC *n* = 22; hIN+mAST *n* = 25; 75 DIV, hIN *n* = 12; hIN+hGPCs *n* = 9; hIN+mAST *n* = 21; 100 DIV, hIN *n* = 7; hIN+hGPC *n* = 8; hIN+mAST *n* = 16, *n* = number of cells; see also Figure S2A). (B) Representative traces of evoked action potentials (APs) triggered by rheobase current injection steps or gradual ramp injection (see also Figures S2F–S2H). (C) Graphs showing AP properties, resting membrane potential (Vrest), AP threshold (APt), AP amplitude (APh), and afterhyperpolarization (AHP) (50 DIV, hIN *n* = 4; hIN+hGPC *n* = 11; hIN+mAST *n* = 20; 75 DIV, hIN *n* = 7; hIN+hGPC *n* = 8; hIN+mAST *n* = 17; 100 DIV, hIN *n* = 4; hIN+hGPC *n* = 7; hIN+mAST *n* = 13, *n* = number of cells). Kruskal-Wallis test at 100 DIV comparing hIN and hIN+mAST *p* = 0.0084 ^∗∗^ (see also Figures S2C and S2D). (D) Percentage and number of cells with AP and no AP across all conditions and time points. Fisher’s exact test at hIN-hIN+mAST *p* < 0.001^∗∗∗^ hIN+hGPC_hIN+mAST *p* < 0.001^∗∗∗^; hIN-hIN+mAST *p* < 0.001^∗∗∗^; hIN-hIN+mAST *p* < 0.001^∗∗∗^; hIN+hGPC-hIN+mAST *p* < 0.001^∗∗∗^. (E) Representative traces of postsynaptic current at 100 DIV and representative trace of postsynaptic current blocked with picrotoxin (PTX) in hIN+mAST condition at 100 DIV. Fisher’s exact test at 50 DIV hIN-hINs+hGPCs *p* < 0.001^∗∗∗^; hIN+hGPC-hIN+mAST *p* < 0.001^∗∗∗^, at 100 DIV; hIN-hIN+mAST *p* < 0.001^∗∗∗^; hIN+hGPC-hIN+mAST *p* < 0.0001^∗∗∗∗^. (F) Percentage and number of cells with postsynaptic activity. *p* < 0.001^∗∗∗^, Fisher’s exact test. (G) Postsynaptic current amplitude for each group (hIN, hIN+hGPC, and hIN+mAST) plotted as cumulative probability. Kolmogorov-Smirnov test at 50 DIV comparing hIN and hIN+hGPC *p* = 0.0483 ^∗^; hIN+hGPC and hIN+mAST *p* = 0.0018 ^∗∗^. Kolmogorov-Smirnov test at 100 DIV comparing hIN and hIN+hGPC *p* < 0.0001 ^∗∗∗∗^; hIN and hIN+mAST *p* < 0.0001 ^∗∗∗∗^; hIN+hGPC and hIN+mAST *p* = 0.0008 ^∗∗∗^. Electrophysiological data were obtained from 2 to 3 independent experiments.

Glial co-cultures have been shown to promote neuronal activity and accelerate neuronal maturation in the differentiation protocol (Purushotham and Buskila, 2023). To assess the neuronal function and maturity as well as the potential effect of co-cultures, we applied whole-cell patch clamp recordings to free-floating spheroids at 50, 75, and 100 DIV (Figure 1A). We first examined the changes in neuronal membrane Na^+^ and K^+^ currents in response to increasing depolarization steps, from −70 to +40 mV (Figure 2A). The activation of voltage-gated Na^+^ and K^+^ channels is necessary for the ability to generate action potentials (APs). Data showed that interneurons expressed voltage-gated Na^+^ and K^+^ channels, with no differences in the magnitude between conditions (Figures 2A and S2A). Similarly, the membrane capacitance remained equivalent among conditions and time points (Figure S2B: 14 ± 2.1–16.6 ± 1.4 at 50 DIV, 17.5 ± 1.3–27.5 ± 4.7 at 75 DIV, and 18.6 ± 2.1–22.5 ± 2.4 at 100 DIV).

To investigate the ability of the cells to elicit APs, we applied stepwise or gradually increased current injections. As shown in Figures 2B and S2F–S2H, cells were capable of firing multiple APs irrespective of time or culture condition. Overall, the AP rate was heterogeneous within and between the groups, indicating a diverse population in neuronal maturation (Figure S2C). Resting membrane potential (Vrest), AP threshold (APt), AP amplitude (APh), and afterhyperpolarization (AHP) did not differ among the groups at 50 and 75 DIV. However, the hIN+mAST group displayed a significantly higher AP amplitude (Figure 2C), and a higher number of cells were able to fire evoked APs (Figure 2D). Upon AP initiation and propagation, neurotransmitters are released at the synaptic terminal, and their binding to postsynaptic ionotropic receptors results in slight changes in the neighboring neuron’s membrane potential that are called postsynaptic currents. Glial cells are crucial in stabilizing neuronal networks by regulating the extracellular concentration of ions and neurotransmitters (Oliveira and Araque, 2022). Therefore, we examined the influence of glial cells on neuronal network activity by recording the frequency and amplitude of postsynaptic currents. At early time points (50 and 75 DIV), all three groups showed similar amplitude and frequency of postsynaptic currents. However, at the later time point (100 DIV), the monocultures of hIN exhibited a higher frequency of postsynaptic currents, or bursting activity, followed by a period of silencing (Figures 2E, 2G, and S2E). This overactivity was not observed in the two co-culture conditions (Figures 2E and 2G), which instead showed lower amplitude of postsynaptic currents in cumulative frequency analysis (100 DIV, Figures 2E, 2G, and S2E), suggesting a more balanced connectivity. Moreover, the postsynaptic currents were entirely blocked with the addition of picrotoxin (PTX), a GABA_A_R antagonist, confirming the GABAergic nature (Figure 2E). The percentage of cells showing postsynaptic currents was comparable across all conditions at 50 and 75 DIV but was significantly higher in glial co-culture at 100 DIV for the hIN+mAST condition (Figure 2F). This suggests higher connectivity and better network function in glial co-cultures, highlighting the importance of glial cells in regulating neuronal activity.

Electrophysiology experiments on H9-derived interneurons at 50 DIV with mAST co-culture showed equivalent current of voltage-gated Na^+^ and K^+^ channels (Figure S2I) and membrane capacitance to RC17-derived neurons (Figure S2J). H9-derived cells further showed Vrest, APt, APh, and AHP like RC17-derived neurons (Figures S2K and S2L).

Overall, these data show an overall neuronal functional maturity and suggest that the addition of astrocytes during spheroid aggregation promotes the formation of more fine-tuned, active neuronal networks.

### Rapid maturation of hESC-derived MGE cells into SST interneurons in 3D spheroids

**Figure 3:**
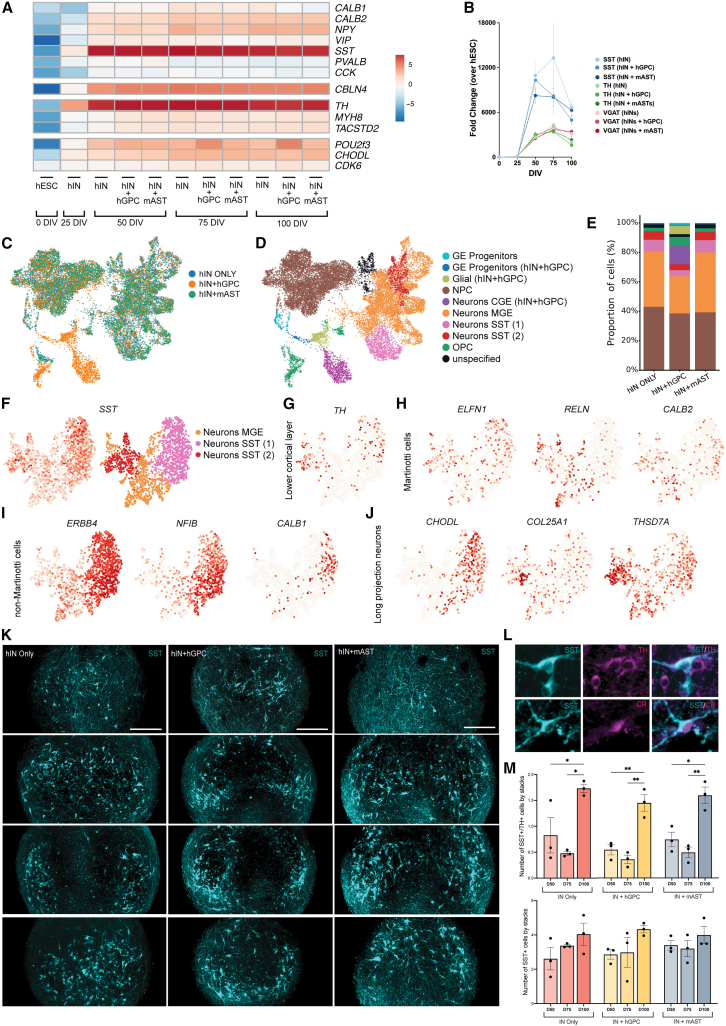
Rapid maturation of hESC-derived MGE cells into SST interneurons in 3D spheroids (A) Heatmap showing relative gene expression levels across the differentiation protocol (see also Table S1 for statistics). (B) Graphs showing the upregulation of *SST*, *VGAT*, and *TH* across time points and conditions, *n* = 4 independent replicates per time point and condition (see also Figures S3A–S3C). (C) UMAP visualizing the cells at 100 DIV. (D) UMAP showing the different clusters across conditions at 100 DIV (see also Figure S3D). (E) Staple bar showing the cell composition of each condition at 100 DIV. (F) UMAP plot showing SST-expressing cells and the clusters of origin across conditions at 100 DIV. (G–J) UMAP plots showing expression of (G) cortical layer-specific markers, (H) Martinotti cell-specific markers, (I) non-Martinotti cell-specific markers and (J) long projection neuron-specific markers on SST population (see also Figure S3E). (K) Z stacks from confocal images, showing the distribution of SST^+^ cells throughout the whole spheroids at 100 DIV (see also Figure S3F). (L) Immunostaining on cryosectioned spheroids, showing the co-localization of SST with CR and TH at 100 DIV (see also Figure S3G). (M) Quantification of SST^+^ cells and SST^+^/TH^+^ cells across (*n* = 3, see also Figure S3H). One-way ANOVA test and post hoc Tukey test. ^∗^*p* < 0.05, ^∗∗^*p* < 0.01. Scale bars: 100 μm.

After confirming the functional activity, we sought to determine the mature neuronal subtypes generated by MGE-like precursors (Figure 1A)*.* We first investigated the molecular profile using qPCR and found significant upregulation of the neuropeptide *SST* at 50 DIV, with expression maintained through 100 DIV (Tables S1C and S1D for statistics; Figures 3A and 3B). This expression followed the induction of *VGAT* (Figure 3B). Moreover, there was significant upregulation in neuropeptide Y and calcium-binding protein *CALB2*, increasing from 50 DIV until 100 DIV (Tables S1C and S1D), indicating gradual induction of these interneuron subtypes (Figure 3A). None of the co-cultures expressed *PVALB*, *CCK*, *CALB1*, and *VIP* at any time point.

Next, we sought to investigate the potential induction of SST subclass genes from different cortical layers (Tasic et al., 2016): upper layer marker *CBLN4*, lower layer markers *TH*, *MYH8*, and *TACSTD2*, and subclasses extending to all cortical layers *CHODL*, *CDK6*, and *POU2F3*. RT-qPCR analysis showed significant induction of *CBLN4* at 50 DIV, particularly in co-culture conditions (Tables S1C and S1D; Figures 3A and S3A), with continuous expression in all groups to 100 DIV (Figures 3A and S3A). *TH* upregulation was observed from 25 DIV and significantly increased at 50 DIV, with the statistical difference in co-culture conditions (Tables S1C and S1D; Figures 3A and 3B). *MYH8* and *TACSTD2* showed modest but significant expression changes, particularly in co-culture conditions (Tables S1A–S1D; Figure S3B). This indicates the potential differentiation into the SST-TH subclass of the lower cortical layer. A lesser, yet significant upregulation of *CHODL* and *POU2F3* was seen at 50 DIV in co-cultures (Tables S1A–S1D; Figures 3A and S3C), with *POU2F3* particularly expressed in the hIN+hGPCs at 100 DIV (Figure S3C). Altogether, these findings suggest a relatively fast (Nicholas et al., 2013) and sustained induction of SST phenotype from 50 DIV in 3D co-cultures, potentially encompassing subclasses of both long-projecting neurons and lower-layer SST cells.

### Single-nuclear RNA sequencing of 3D spheroids reveals distinct subclasses of SST interneurons

To further confirm and characterize the SST population and unravel other potential neuronal subtypes, we performed single-nuclear RNA sequencing (snRNA-seq). We selected samples at 100 DIV based on the stable gene expression of *SST*, *VGAT*, and *TH* at this time point (Figure 3B). Integration of sequencing data from the three groups revealed similar cell composition (Figures 3C and 3E). The one exception was a separate cluster derived from the hIN+hGPC group (Figures 3C and 3E, orange cluster). Clusters were characterized as distinct cell phenotypes according to their gene expression patterns (Figure 3D). A dotplot, as presented in Bershteyn et al. (2024), showed that ganglionic eminence (GE) progenitors were depicted by GE progenitor markers (*MKI67*, *TOP2A*, *PCLAF*, and *ASPM*) and were also positive for MGE (*SOX6*), pallium (*ZEB2* and *ERBB4*), and sub-pallium genes (*PBX3*). Neural progenitor cells (NPCs) were positive for NPC markers (*NKX2-1*, *VIM*, *OTX2*, and *PTPRZ1*) but also positive for *SOX6* and pallial markers (Figure S3D). Neurons of the CGE expressed CGE characteristic genes (*SCGN*, *CALB2*, *PROX1*, and *KLHL35*), whereas the MGE cluster expressed MGE-specific genes (*SOX6*, *LHX6*, *NXPH1*, and *PDZRN4*) in addition to neuronal and GABAergic genes (Figure S3D). The neurons of the SST1 cluster showed gene induction of neurons, GABAergic, MGE (specifically *SOX6*, and *NXPH1*), and pallial genes (*NXPH1*, *ERBB4*, and *SST*), whereas neurons of the SST2 cluster expressed genes of neurons, GABAergic, MGE, and sub-pallium (*NRP2*, *PBX3*, and *ISL1*) (Figure S3D).

Further integration of the SST^+^ cells revealed three distinct subgroups, corresponding to the previously established SST1, SST2, and MGE clusters, with similar gene expression profiles (Figures 3F and S3D). The SST^+^ cells expressed *TH*, suggesting the presence of this lower cortical layer subclass (Tasic et al., 2016) (Figure 3G). While genes that are characteristic of Martinotti cells, such as *ELFN1*, *RELN*, and *CALB2*, were expressed evenly throughout the clusters (Figure 3H), the non-Martinotti genes, *ERBB4* and *NFIB*, were mainly found in the SST1 cluster (Figure 3I). Genes associated with long-projection neurons, *CHODL*, *COL25A1*, and *THSD7A*, were expressed in all three *SST*^+^ clusters (Figure 3J). While upper cortical layer marker *CBLN4* and pan-layer marker *POU2F3* were not seen, there were some expressions of *CDK6* (Figure S3E). Taken together, this data show gene induction of distinct SST interneuron subclasses in 3D long-term cultures.

### Immunocytochemistry of 3D spheroids confirms distinct subclasses of SST interneurons

To validate the distinct upregulation of *SST* expression, we performed immunofluorescent analysis and found abundant SST protein distributed throughout the entire spheroids as early as 50 DIV (Figure S3F) and up to 100 DIV in all groups (Figure 3K). Higher magnification demonstrated a mature neuronal morphology for SST neurons already seen at 50 DIV (Figure S3G) and up to 100 DIV (Figure 3L). Importantly, SST neurons were present in the middle of the spheroids, indicating no necrotic core or incomplete differentiation, as sometimes observed in larger 3D organoids (Bhaduri et al., 2020). Quantifications showed a comparable number of SST^+^ cells across the hIN, hIN+hGPC, and hIN+mAST conditions with a trend of increase to 100 DIV (Figure 3M, 0.2–1.5 cells/stacks with 120–130 stacks/spheroids, see methods). Interestingly, we observed co-localization of TH or calretinin (CR) protein (encoded by *CALB2* gene), suggesting the presence of these subclasses (Figure 3L). SST-TH^+^ subtype showed a significant increase at 100 DIV (Figure 3M), whereas neurons positive for SST+CR were lower in number compared to TH, and similar across conditions (Figure S3H, 0.2 cells/stack). In conclusion, these results align with the RT-qPCR and snRNA-seq data, confirming a rapid maturation into SST interneurons that is similar between the co-culture conditions. Moreover, it supports long-term culture as a mean to increase the yield for the SST-specific subclass.

RT-qPCR data from the experiment with the H9 cells also showed upregulation of *SST* and *TH* at 50 DIV in co-culture with mAST (Figure S3I). At this time point, we could also detect SST^+^ cells throughout the spheroid (Figure S3J), some of them co-expressing TH (Figure S3K). The number of SST^+^ and SST^+^/TH^+^ cells in the spheroid was similar to the RC17-derived interneurons with mAST (Figure S3L).

Overall, these results demonstrate a rapid and stable differentiation into SST interneurons in a 3D structure that is independent of glial co-culture and stem cell line. The SST fate specification shows subclass-specific gene and protein expression, including that of long-projecting neurons and the SST-TH subclass that increases up to 100 DIV.

### hESC-derived interneuron subtypes mature and functionally integrate in a cortical organoid circuit

**Figure 4:**
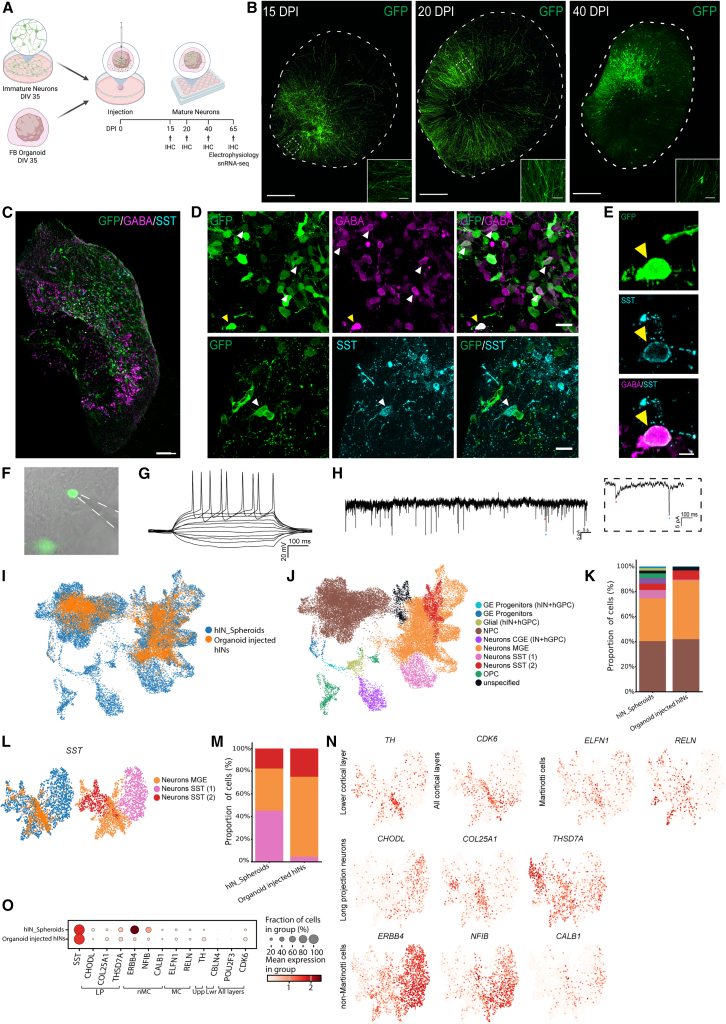
hESC-derived interneuron subtype maturation and functional integration in an artificial cortical circuit (A) Schematic overview of injection of MGE-like progenitor cells into human FB organoids. (B) Images of FB organoids, injected with GFP^+^ MGE-like progenitors at 15, 20, and 40 DPI (endogenous GFP fluorescence, see also Figure S4A; scale bars, 500 μm). (C) Immunohistochemistry of cryosectioned FB organoids injected with GFP^+^ MGE-like at 65 DPI. Scale bars, 100 μm. (D) Immunohistochemistry showing double-positive cells for GFP-GABA and GFP-SST at 65 DPI. Scale bars, 20 μm. (E) Immunohistochemistry showing co-localization of GFP with GABA and SST at 65 DPI. Scale bars, 50 μm. (F) Patched GFP^+^ cell at 65 DIV. (G) Representative traces of evoked APs triggered by rheobase current injection steps (see also Figure S4B). (H) Representative traces of postsynaptic activity of patched cell and higher magnification. (I) UMAP plot visualizing general overlap of hIN spheroids (blue, all conditions) and organoid-injected hIN at 100 DPI. (J) UMAP plot showing the different clusters for both spheroid cells and organoid-injected GFP^+^ cells (see also Figure S4C). (K) Staple bar of cell composition in spheroids (all conditions at 100 DIV) contra organoid-injected GFP^+^ cells (100 DPI, *p* < 0.001 for hIN spheroid vs. organoid-injected hINs, chi square test). (L) UMAP plot for the SST cells derived from hIN spheroids (blue) and organoid-injected hIN (yellow) together with clusters of origin. (M) Staple bar showing the proportion of SST-expressing cells derived from hIN spheroid and organoid-injected hIN, (*p* < 0.001 for SST1, SST2, and MGE group). (N) UMAP plots on SST population of hIN spheroid and organoid-injected hIN showing the expression of cortical layer-specific markers, Martinotti cell-specific markers, long-projection neuron-specific markers, and non-Martinotti cell-specific markers (see also Figures S4D and S4E). (O) Dot plot showing the co-expression of *SST* with subclass-specific markers.

Having established that our MGE-patterned progenitors can mature into subclass-specific SST interneurons in a spheroid system, we wanted to explore if they could functionally integrate within a human cortical network and if their subtype specificity was influenced by the FB environment of both excitatory and inhibitory neurons. To this end, GFP-expressing MGE-like progenitors labeled with cytoplasmic or nuclear GFP were injected at 35 DIV into human FB organoids (Sozzi et al., 2022) (Figures 4A and S4A). The organoids were of the same developmental age, i.e., 35 days, corresponding to the peak period of deep-layer pyramidal neurons, which is crucial for proper migration of MGE-derived interneurons (Lodato et al., 2011; Pollen et al., 2019). Imaging of the endogenous GFP signal in organoids at 15–20 days post injection (DPI) showed widespread migration of the MGE-like progenitors from the injection site in the FB organoid toward the periphery, displaying complex morphology and long projections (Figure 4B). At 40 DPI, the GFP interneurons showed an even more evident peripheral localization (Figure 4B). Immunocytochemistry of GFP, GABA, and SST at 65 DPI showed widespread distribution of these neuronal subtypes in the human organoid (Figure 4C) and importantly co-localization of GFP and GABA or GFP and SST (Figure 4D) and sometimes both proteins, i.e., SST and GABA (Figure 4E). This demonstrates that the MGE-derived interneurons survive and integrate into the human FB environment for up to 100 DIV with similar SST expression.

Whole-cell patch clamp electrophysiology was applied at 65 DPI (corresponding to 100 DIV) for examining functional maturity and possible synaptic integration into the native network (Figure 4F). Injected GFP^+^ cells displayed inward Na^+^ and outward K^+^ currents of similar amplitude as in the aforementioned 3D settings (Figure S4B compared to Figure 2A) and were able to induce APs in response to current injection, supporting their neuronal maturity (Figure 4G, *n* = 8 cells). Importantly, postsynaptic activity (Figure 4H) showed events of both slow-decaying GABAergic shape and fast-decaying glutamatergic shape that were not previously seen in the hIN spheroid condition. This suggests that MGE progenitors not only functionally mature in FB organoids but also receive excitatory and inhibitory synaptic input, partly from the human FB neurons.

### snRNA-seq of injected MGE-like progenitors reveals a bias toward subpallial SST subtypes

To assess the SST population and unravel other potential neuronal subtypes in the organoid-injected cells, we performed snRNA-seq at 65 DPI (corresponding to 100 DIV) on fluorescence-activated cell sorting (FACS)-sorted GFP^+^ nuclei. An integrated uniform manifold approximation and projection (UMAP) analysis of the organoid-injected cells versus the spheroids (all three groups) demonstrated similar composition and gene expression profile, except for the CGE purple cluster, oligodendrocyte precursor cell (OPC) green cluster, and glial light green cluster (Figures 4I and 4J). A comparative proportion analysis showed similar levels of NPC but an increased proportion of MGE cells positive for *SOX6* and *NXPH1* in the organoids (Figures 4K and S4C). Organoid-injected cells further showed little or no expression of glial, OPC, or GE progenitor genes (Figure S4C) but increased SST2 fate (labeled in red, *p* < 0.001), characteristic of subpallial SST neurons, and MGE fate (labeled in yellow, *p* < 0.001, Figures 4L and 4M). SST cells in organoids thus belonged to SST2 and MGE groups (labeled in red and yellow, Figure 4L) with TH co-expression, similar to when in spheroid culture (Figure 4N for spheroid and organoid; Figure S4E for organoid only). In addition, organoid SST cells expressed genes typical of Martinotti (*ELFN1*, *RELN*, and *CALB2*, Figure 4N) and long-projecting neurons (*COL25A1* and *THSD7A*, Figure 4N), with low expression of non-Martinotti markers (*ERBB4*, *NFIB*, and *CALB1*, Figures 4N and S4E). A dotplot on the SST-expressing cells showed similar fractions of marker genes for Martinotti cells and long-projecting neurons in the spheroid and organoid but lower expression of non-Martinotti genes (especially for the *ERBB4* and *NFIB*) in the organoid (Figures 4O, S4C, and S4E for organoid only).

Altogether, this shows that while SST interneuron phenotype persists in the FB organoid, the environmental cues appear to affect the subclasses toward the SST subpallial group in contrast to the more diverse subclass seen in the spheroid. Interestingly, the long-projecting SST subclass remains in equal proportion in both environments.

## Discussion

Cortical (pallial) GABAergic interneurons are the primary source of inhibition in the cortex and hippocampus and are originally generated in distinct germinal zones in the developing brain. MGE-derived PV and SST-expressing interneurons have shown specific importance in the physiological signaling regulation, with their dysfunction linked to several neurological disorders (Batiuk et al., 2022; Pfisterer et al., 2020). Therefore, it is instrumental to derive functional human FB GABA interneurons, especially those from people affected with interneuron deficit disease, for the investigation of disease pathogenesis and development of therapeutics. The ability to generate MGE interneurons that closely resemble bona fide human interneurons has made some progress in recent years (Bershteyn et al., 2023). Nevertheless, the derivation of cortical interneurons *in vitro* remains challenging, potentially due to the extended maturation time and synaptic support needed for these cells to differentiate (Close et al., 2017; Maroof et al., 2013; Nicholas et al., 2013).

In this study, we differentiated hESCs into subtype-specific SST interneurons using a 3D co-culture platform. Under these conditions, hESCs first differentiated to MGE progenitors and began expressing neuronal markers at 50 DIV and beyond, with a notable upregulation of *DLX5* and *NKX2-1*, key regulators of cortical interneuron development and migration (Maroof et al., 2013). Importantly, the 3D culture environment supported rapid and stable differentiation into SST interneurons, with subclass specification observed as early as 50 DIV, a faster process compared to previous stem cell-derived protocols in conventional 2D cultures (Nicholas et al., 2013). While SST interneurons have been derived *in vitro* previously, subclass specification has primarily been investigated in rodent models (Gouwens et al., 2020; Tasic et al., 2016, 2018; Wu et al., 2023) with limited exploration in hESC-derived systems (Bershteyn et al., 2024). Therefore, our 3D model provides valuable insights into SST subclasses in a human *in vitro* system.

Importantly, we herein detected the transcriptomic signature of the long-projection SST neuronal subtype (*SST-CHODL* and *SST-THSD7A*). These are specialized subtypes of GABAergic neurons that extend across different cortical layers and are among the most conserved subtypes of the mammalian neocortex, with important implications for cortical function (Callaway et al., 2021; Fisher et al., 2024). In addition to the long-projection neurons, our spheroid differentiation protocol gave rise to SST cells expressing genes related to both upper (*SST-CBLN4*) and lower cortical layers (*SST-CALB2*, *SST-TH*, and *SST-CALB1*) (Tasic et al., 2016), corresponding to both Martinotti and non-Martinotti subtypes (Fisher et al., 2024; Tasic et al., 2016). Martinotti cells are the most abundant SST subclass, defined by their axonal plexus in L1 or axons that ramify in both L2/3 and L1 or L1 alone, while non-Martinotti cells target L4 instead of L1 (Ma et al., 2006; Xu et al., 2013). Recently, single-cell genomics has expanded the knowledge of interneuron diversity and revealed additional SST subtypes, including *SST-TH* and *SST-CDK6* (Mayer et al., 2018; Paul et al., 2017; Tasic et al., 2016). While the importance of these subclasses remains to be fully explored, our protocol could aid in further investigation within a human context.

The integration of exogenous cell types into brain organoid circuits has recently explored extrinsic effects on neuronal maturation and axonal projections (Reumann et al., 2023; Sabate-Soler et al., 2022). Here, we followed a similar approach, injecting hESC-derived interneurons into FB organoids to investigate how cellular interactions influenced subtype specification and functional maturity. We observed that the injected cells migrated throughout the entire organoid structure within only 20 days, functionally integrating into the host circuits through active synapses. The organoid environment favored the SST2 cluster, corresponding to a more distinct subpallial fate with lower expression of non-Martinotti genes (e.g., *ERBB4* and *NFIB*). These genes, which are also markers of immature migrating interneurons, suggest better maturation of the SST interneurons in the organoid environment, supporting previous *in vivo* studies (Upadhya et al., 2019). Interestingly, SST cells expressing genes of long-range projection neurons (*SST-CHODL*, *COL25A1*, and *POU2F3*) remained upon organoid injection despite the larger size of the 3D structure and the projection potential. These observations suggest that while the diversification of some SST^+^ interneuron subtypes may require additional signals beyond those intrinsically programmed during embryonic development, the long-range projection neuron subclass is defined early in embryonic development and is less influenced by environmental signals (Bershteyn et al., 2024; Fisher et al., 2024). Moreover, our data support previous reports showing various timing of interneuron diversification among different SST^+^ neurons (Fisher et al., 2024; Yu et al., 2021).

In line with previously published studies using similar protocols, we did not observe any PV^+^ interneurons throughout our differentiation timeline in any of the co-cultured spheroids or injected organoids (Close et al., 2017). It is possible that a more heterogeneous environment, including glutamatergic input from early differentiation stages, is required to generate PV^+^ neurons, as this subtype is sensitive to intrinsic factors, connectivity, and attractor dynamics (De Marco Garcia et al., 2011; Fishell and Kepecs, 2020; Nicholas et al., 2013; Ruden et al., 2021).

We co-cultured hESC-derived interneurons with different glial cells to improve functional maturity and network connectivity. However, functional analysis from cultures with or without glia demonstrated similar neuronal maturity and inhibitory network connectivity at 50 and 75 DIV, suggesting no additional support from glial progenitor cells or astrocytes during the initial phase. It was not until 100 DIV that the co-cultures functionally supported network connectivity by preventing burst-like activity, likely through neurotransmitter buffering (Purushotham and Buskila, 2023). Notably, co-culture with astrocytes appeared to increase functional maturity and network connectivity, by a higher proportion of cells firing and forming postsynaptic connections. This finding confirms the supportive role of astrocytes in neuronal maturity and underscores the applicability of mouse astrocytes to support human neuronal cultures (Canals et al., 2018; Enright et al., 2020). Future studies on long-term 3D cultures should incorporate astrocyte co-culture to improve neuronal function and connectivity.

In conclusion, we herein provide a culture system for the long-term maintenance of glia-interneuron interactions in both healthy and pathological development. Our 3D culture model enables the derivation of human SST-specific phenotypes with subclass specificity, a feature not previously demonstrated in human pluripotent stem cell-based protocols (Close et al., 2017). While our spheroid culture system maintains a homogeneous inhibitory circuit at a functional level, it cannot mimic the range of cues from early network activity to cortical pyramidal function (De Marco Garcia et al., 2011; Lodato et al., 2011; Wester et al., 2019). However, the successful integration of interneurons into the artificial cortical circuit of FB organoids offers promising opportunities for more complex disease modeling in future.

## Methods

Detailed descriptions of the experimental procedures can be found in the supplemental information.

### Cell culture

Human interneuron differentiation was initiated from the hESC lines RC17 (RCe021-A, p35-40) and H9 (WAe009-A, p42). hESCs were expanded in 6-well plates coated with LN521 (0.5 mg/cm^2^; BioLamina, Sundbyberg, Sweden) in iPS Brew XF medium (Miltenyi Biotec, Bergisch Gladbach, Germany). Cells were passaged at 80% confluency using EDTA (0.5 mM; MA, USA). During the passage, cells were replated into new LN521-coated 6-well plates in iPS Brew XF medium supplemented with the Rho-associated protein kinase inhibitor (Y27632; STEMCELL Technologies, British Columbia, Canada). After two passages, the interneuron differentiation protocol adapted from Nicholas et al. (2013) was initiated according to Figure 1A. hGPCs were generated from hESCs (RC17 Roslin cells, cat. no. hPSCreg RCe021-A, p40–45), according to published protocols (Nolbrant et al., 2020; Wang et al., 2013). MGE-like progenitors were transduced at 25 DIV with a lentiviral construct carrying GFP for cytoplasmic localization and nuclear GFP for subsequent nuclei extraction. FB organoids were generated as previously described (Lancaster et al., 2017; Sozzi et al., 2022).

### Immunofluorescent staining, high-content screening, and microscopy

Immunofluorescent staining was performed on spheroids at 35, 50, 75, and 100 DIV, as well as organoids at 65 DPI. Cells and spheroids were fixed with 4% PFA for 10–15 min at room temperature (RT), and organoids were fixed with 4% PFA overnight at 4°C. For full spheroid and organoid staining, we adapted a previously described protocol (Giacomoni et al., 2023). High-content screening was performed for analysis at 35 DIV using Operetta CLS (PerkinElmer). Fluorescent microscopy images were captured using either a Leica Stellaris 5 or Zeiss 780 confocal lase -scanning inverted microscope and processed using the LAS X (Leica) or ZEN (Zeiss) software, respectively. Image adjustments were applied equally across all images without loss of information.

### Electrophysiology

Spheroids at 50, 75, and 100 DIV were analyzed using whole-cell patch-clamp recordings. Free-floating spheroids were placed in the recording chamber with constant perfusion of Krebs solution, gassed with 95% O_2_–5% CO_2_ at RT, during recording. The composition of the Krebs solution was (in mM) 119 NaCl, 2.5 KCl, 1.3 MgSO_4_, 2.5, 1.25 mM NaH_2_PO_4_ CaCl_2_, 25 glucose, and 26 NaHCO_3_. Recordings were made using a Multiclamp 700B amplifier (Molecular Devices, San Jose, CA, USA) with pulled borosilicate glass pipettes (3–7 MOhm) filled with the following intracellular solution (in mM): 122.5 potassium gluconate, 12.5 KCl, 0.2 EGTA, 10 HEPES, 2 MgATP, 0.3 Na_3_GTP, and 8 NaCl adjusted to pH 7.3 with KOH. Data acquisition was performed with pClamp 10.2 (Molecular Devices, San Jose, CA, USA). The current was filtered at 0.1 kHz and digitized at 2 kHz.

### Nuclei isolation from spheroids, isolation of GFP^+^ cells from the organoid, and FACS-based sorting

Spheroids and organoids were collected at 100 DIV, snap-frozen, and stored at −80°C until nuclei extraction. Spheroid nuclei isolation was performed according to the following protocol (Södersten et al., 2018) with modifications (Fiorenzano et al., 2024).

### snRNA-seq and analysis

Single-nuclei suspensions were loaded onto 10× Genomics Single Cell 3′ Chips (v.3.1), and single-nuclei gel beads in emulsion (GEMs, v.3 chemistry) were generated following the manufacturer’s protocol (https://support.10xgenomics.com/single-cell-gene-expression/index/doc/technical-note-chromium-single-cell-3-v3-reagent-workflow-and-software-updates). The 10× libraries were sequenced on a NovaSeq 6000 with the following steps: 28 cycles of Read1, 98 cycles of Read2, and 8 cycles of Index1, using a 200-cycle kit. Raw base calls were demultiplexed and converted to FASTQ files for downstream analysis using the cellranger mkfastq pipeline (bcl2fastq 2.20/cellranger 6.0). The raw snRNA-seq data were processed using Cell Ranger (v.7.1.0) and annotated with the GRCh38-2020 human reference transcriptome from 10× Genomics. Subsequent analysis was performed in Python (v.3.10.12) using Scanpy (v.1.9.5). Quality control was conducted separately on the five initial datasets (three datasets from spheroids at 100 DIV and two datasets from organoid-injected cells at 100 DIV), filtering out nuclei with fewer than 200 genes or more than 1% mitochondrial genes. Moreover, genes detected in fewer than three nuclei, mitochondrial genes, *MALAT1* genes, and hemoglobin genes were excluded (see Figures S4F–S4I). The datasets were normalized to 10,000 counts per nucleus, log-transformed, and scaled. Doublet nuclei were identified with Scrublet (v.0.2.3) and removed, followed by re-normalization.

### Quantification, data presentation, and statistical analysis

All data are presented as mean ± SEM. Statistical analysis for cell quantification and electrophysiology data was performed using one-way analysis of variance (ANOVA) followed by post hoc Tukey in GraphPad Prism 10.4.2 (GraphPad, San Diego, CA, USA); Fisher’s exact test was used to compare proportions, unless otherwise stated. Statistical analysis for RT-qPCR data was performed in R (version 4.3.3) using the *stats* package, unless specified otherwise. The Shapiro-Wilk normality test was performed, and subsequently, parametric ANOVA with post hoc Tukey test (Hothorn et al., 2008) and non-parametric tests (Kruskal-Wallis with post hoc Dunn’s test [version 0.9.5]) were done accordingly. The *p* values reported for PCR data are from 4 different independent experiments (i.e., independent replicates). *p* values for electrophysiological data and quantifications were obtained from 2 to 3 independent replicates. For Figures 4K and 4M, a chi-square test was performed.

## Resource availability

### Lead contact

Requests for further information and resources should be directed to and will be fulfilled by the lead contact, Daniella Rylander Ottosson (daniella.ottosson@med.lu.se).

### Materials availability

This study did not generate new, unique reagents.

### Data and code availability

The accession number for the snRNA seq data reported in this paper is NCBI GEO: GSE305121.

## Acknowledgments

This work was supported by several core facilities funded by Lund Stem Cell Center and MultiPark. We would like to thank Malin Parmar for her scientific input and support and resources, Anna Hammarberg for her assistance with nuclei sorting, Jenny Johansson for cDNA library preparation, Emanuella Monni for her assistance with confocal imaging, and Ulla Jarl for assisting in tissue preparation for histology. Graphical schematics were created using BioRender.com. We acknowledge the following funders for this project: 10.13039/501100004359Swedish Research Council 2021-01839 and 2021-03149 (D.R.O.), 10.13039/501100004063Knut and Alice Wallenberg Foundation 2021-0088 (D.R.O.) Crafoord Foundation 20231012 (D.R.O.), Jeansson Foundation JS2018-0103, The Swedish Brain Foundation FO2019-0195 (D.R.O.), Åhléns foundation 139208 (D.R.O.), Royal Physiographical Society and Per-Eric Ulla Schyberg Foundation, Sweden (E.C.-P., 43202; 45610, D.R.O), Olle Engkvist Foundation 213-0229 (D.R.O. and E.C.-P.), Anna-Lisa Rosenberg Foundation (E.C.-P.), and Royal Physiographic Society in Lund 43202 (D.R.O.).

## Author contributions

A.B.: investigation, formal analysis, methodology, validation, visualization, conceptualization, and writing – original draft. C.-A.S.: formal analysis, investigation, validation, visualization, and writing – review annd editing. A.-L.H.: data curation, investigation, formal analysis, validation, visualization, and writing – review and editing. C.A.-M.: formal analysis, visualization, and writing – review and editing. E.C.-P.: data curation, formal analysis, investigation, validation, visualization, and writing – review and editing. E.S.: resources, investigation, and writing – review and editing. G.R.P.: formal analysis, investigation, and visualization. G.N.: formal analysis and visualization. J.G.: resources, investigation, and writing – review and editing. V.O.: data curation, formal analysis, methodology, supervision, validation, visualization, and writing – review and editing. D.R.O.: conceptualization, funding acquisition, project administration, visualization, resources, supervision, and writing – original draft, review and editing.

## Declaration of interests

The authors declare no competing interests.

## Supplemental information

Document S1. Figures S1–S4, Tables S2–S4, and supplemental methods

Table S1. Statistical analysis of gene expression measured by qPCR Values reported are p-values between the given groups and time points from 4 independent experiments (replicates).

Document S2. Article plus supplemental information
