# Supplementary material for: Three-dimensional co-culturing reveals human stem cell-derived somatostatin interneurons with subclass expression
Source: Stem Cell Reports. 2025 Sep 9;20(9):102634. doi: 10.1016/j.stemcr.2025.102634 (PMC12447333; doi:10.1016/j.stemcr.2025.102634)
Supplement: Document S1. Figures S1–S4, Tables S2–S4, and supplemental methods [file mmc1.pdf]

Stem Cell Reports, Volume *20*

Supplemental Information

Three-dimensional co-culturing reveals human stem cell-derived somatostatin interneurons with subclass expression

Andreas Bruzelius, Christina-Anastasia Stamouli, Anna-Lena Hölldobler, Constanza Aretio-Medina, Efrain Cepeda-Prado, Edoardo Sozzi, Germán Ramos Passarello, Gianluigi Nocera, Jessica Giacomoni, Victor Olariu, and Daniella Rylander Ottosson

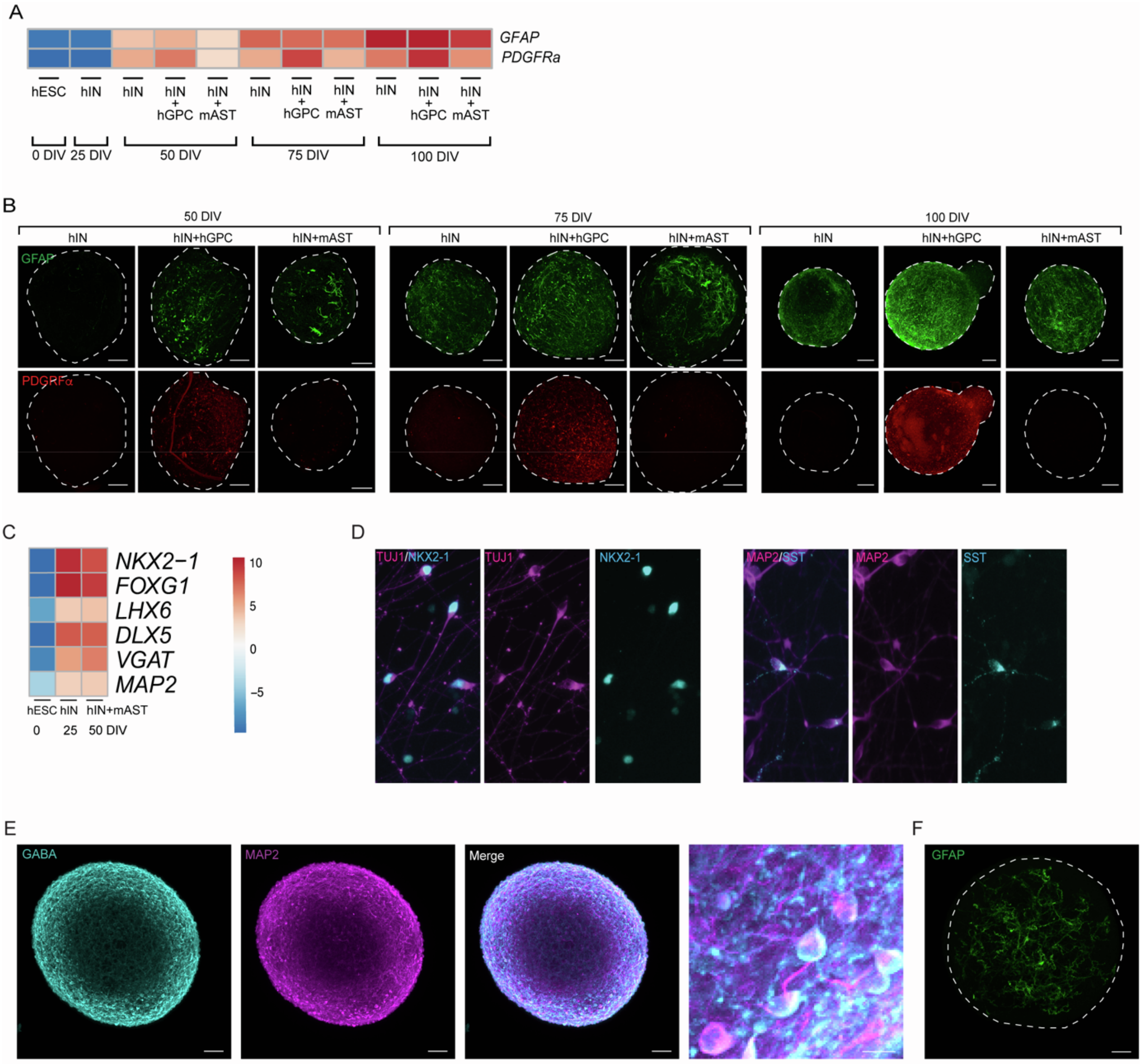

Figure S1. Characterization of glial cells in 3D co-cultures and differentiation of H9 cells line into GABAergic interneurons in 3D co-culture. Related to Figure 1. A) Heatmap showing relative gene expression levels of different time points across a differentiation protocol. **B)** Maximum intensity projection of confocal images of GFAP and PDGFRa expression across conditions and time points. Scale bar = 100 µm. **C)** Heatmap showing relative gene expression levels of different time points across a differentiation protocol with H9 cells. **D)** Immunofluorescence showing expression of NKX 2.1, TUJ1, MAP2, SST, and GABA at 35 DIV of H9 cells. **E)** Maximum intensity projection images showing MAP2 and GABA expression throughout the whole spheroid volume at 50 DIV of H9 cells. Scale bar = 100 µm **F)** Maximum intensity projection images of GFAP at 50 DIV. Scale bar = 100 µm. hESC= human embryonic stem cells; hIN= human interneurons; hGPC= human glial progenitor cells; mAST= mouse astrocytes; DIV= days *in vitro*.

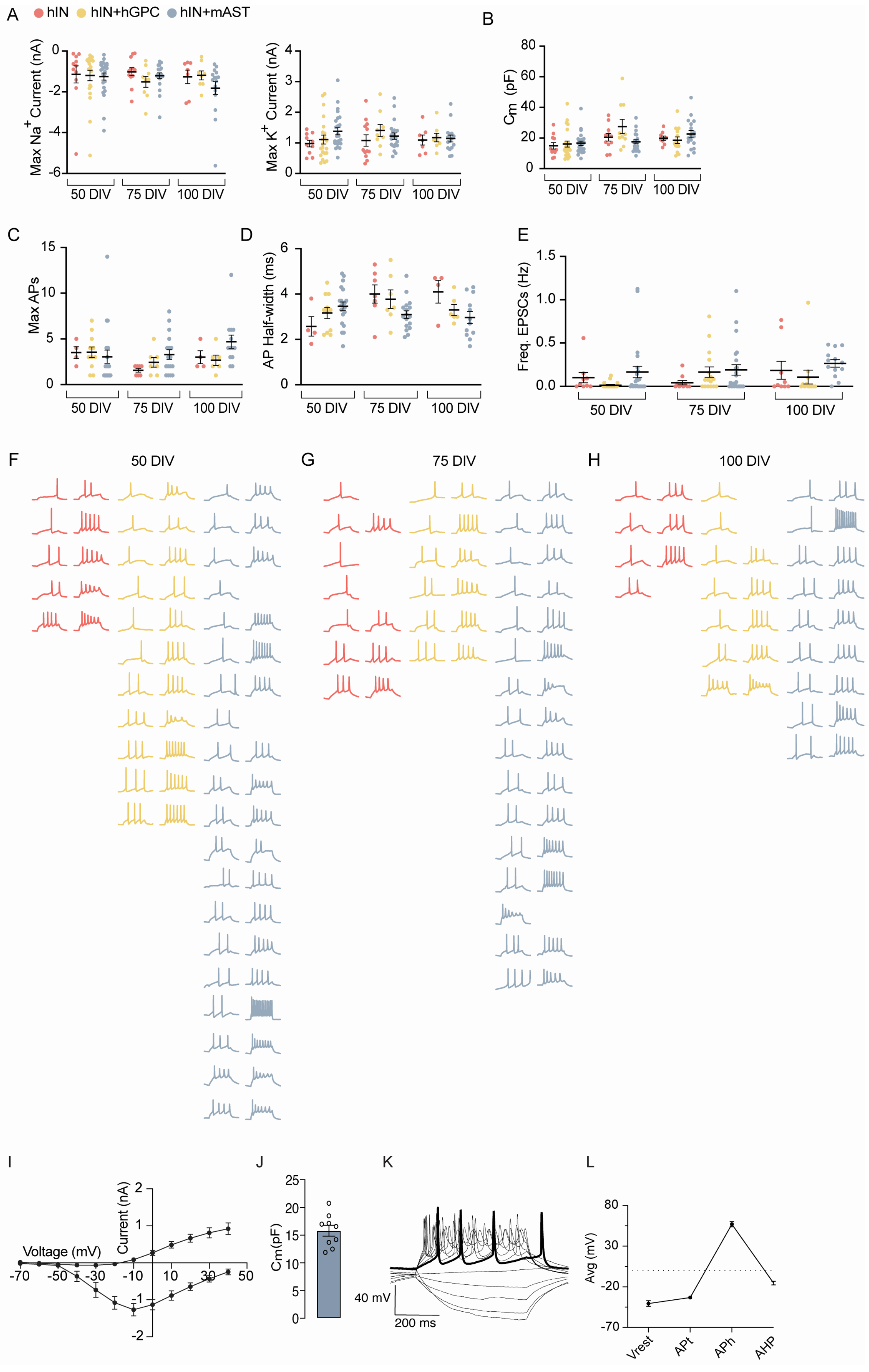
A
hIN
hIN+hGPC
hIN+mAST
Max Na+ Current (nA)
Max K+ Current (nA)
50 DIV
75 DIV
100 DIV
B
Cm (pF)
C
Max APs
D
AP Half-width (ms)
E
Freq. EPSCs (Hz)
F
G
H
I
Voltage (mV)
Current (nA)
J
Cm(pF)
K
40 mV
200 ms
L
Avg (mV)
Vrest
APt
APh
AHP

Figure S2. Electrophysiological properties of 3D co-cultures across conditions and time points and H9-derived co-culture. Related to Figure 2. A) Maximum Na^+ and maximum K^+ current displayed by cells at all timepoints and across conditions. **B)** Membrane capacitance of recorded cells at all timepoints and across conditions. **C)** Max action potentials (APs). **D)** Half-width of the first evoked AP. **E)** Frequency of the excitatory post-synaptic potentials (EPSPs) across all conditions and time points. Maximum AP displayed by each cell across all conditions and time points. **F-H)** Traces of the patched cells across all conditions at **F)** 50 DIV, **G)** 75 DIV and **H)** 100 DIV. Electrophysiology data were obtained from 2-3 independent experiments. **I)** Inward Na+ and outward K+ current plotted against voltage injection for hIN + mAST at 50 DIV of H9 cells. **J)** Membrane capacitance of recorded cells for hIN + mAST at 50 DIV of H9 cells. **K)** Representative traces of evoked action potentials (AP) for hIN + mAST at 50 DIV of H9 cells. **L)** Graphs showing AP properties across time points and conditions, resting membrane potential (Vres), AP threshold (APt), AP amplitude (APh) and afterhyperpolarization (AHP) at 50 DIV of H9 cells. (n=9). DIV=days *in vitro*; hIN=human interneurons; hGPC=human glial progenitor cells; mAST=mouse astrocytes.

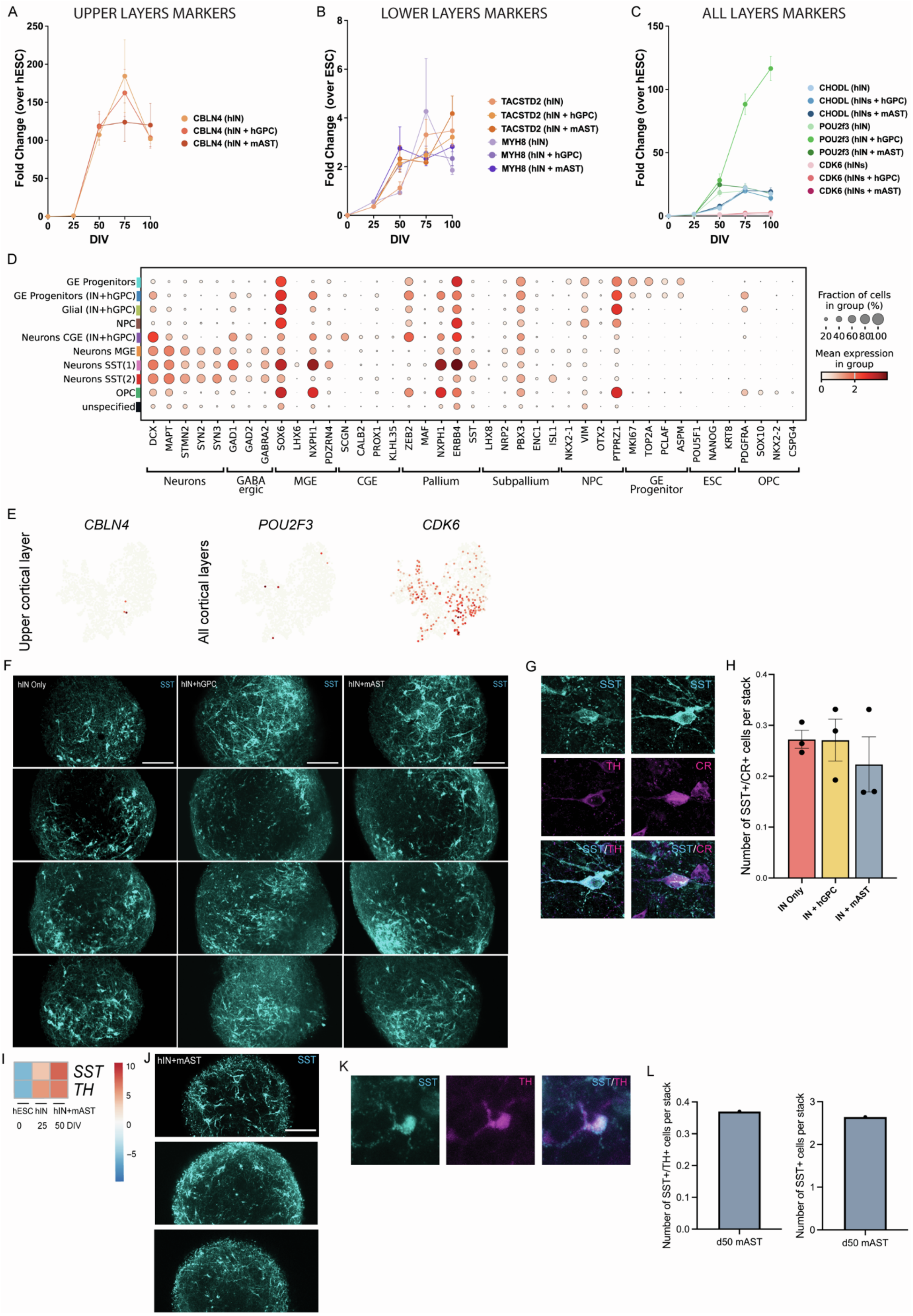

A
UPPER LAYERS MARKERS
Fold Change (over hESC)
DIV
CBLN4 (hIN)
CBLN4 (hIN + hGPC)
CBLN4 (hIN + mAST)
B
LOWER LAYERS MARKERS
Fold Change (over ESC)
DIV
TACSTD2 (hIN)
TACSTD2 (hIN + hGPC)
TACSTD2 (hIN + mAST)
MYH8 (hIN)
MYH8 (hIN + hGPC)
MYH8 (hIN + mAST)
C
ALL LAYERS MARKERS
Fold Change (over hESC)
DIV
CHODL (hIN)
CHODL (hINs + hGPC)
CHODL (hINs + mAST)
POU2f3 (hIN)
POU2f3 (hIN + hGPC)
POU2f3 (hIN + mAST)
CDK6 (hINs)
CDK6 (hINs + hGPC)
CDK6 (hINs + mAST)
D
GE Progenitors
GE Progenitors (IN+hGPC)
Glial (IN+hGPC)
NPC
Neurons CGE (IN+hGPC)
Neurons MGE
Neurons SST(1)
Neurons SST(2)
OPC
unspecified
DCX MAPT STMN2 SYN2 SYN3 GAD1 GAD2 GABRA2 SOX6 LHX6 NXPH1 PDZRN4 SCGN CALB2 PROX1 KLHL35 ZEB2 MAF NXPH1 ERBB4 SST LHX8 NRP2 PBX3 ENC1 ISL1 NKX2-1 VIM OTX2 PTPRZ1 MKI67 TOP2A PCLAF ASPM POU5F1 NANOG KRT8 PDGFRA SOX10 NKX2-2 CSPG4
Neurons
GABA ergic
MGE
CGE
Pallium
Subpallium
NPC
GE Progenitor
ESC
OPC
Fraction of cells in group (%)
20 40 60 80 100
Mean expression in group
0 2
E
Upper cortical layer
CBLN4
All cortical layers
POU2F3
CDK6
F
hIN Only
hIN+hGPC
hIN+mAST
SST
G
SST
TH
CR
SST/TH
SST/CR
H
Number of SST+/CR+ cells per stack
IN Only
IN + hGPC
IN + mAST
I
SST
TH
hESC hIN hIN+mAST
0 25 50 DIV
J
hIN+mAST
SST
K
SST
TH
SST/TH
L
Number of SST+/TH+ cells per stack
d50 mAST
Number of SST+ cells per stack
d50 mAST

Figure S3. Subtype and subclass marker expression across conditions and time points and H9-derived interneurons. Related to Figure 3. A-C) Graphs showing the upregulation of A) upper layer markers, B) lower layer markers and C) all layer markers across all conditions and time points. **D)** Dot plot showing the expression of genes representative for each cluster of Figure 3D. Dot plot is similar to the format presented in Bershteyn et al., 2024. **E)** UMAP plots showing the expression of CBLN4, POU2F3 and CDK6 on SST population. **F)** Z-stacks from confocal imaging, showing the distribution of SST+ cells throughout the whole spheroids at 50 DIV across conditions. Scale bar=100 µm **G)** Immunostaining on cryosectioned spheroids, showing the co-localization of SST with CR and SST with TH at 50 DIV. **H)** Quantification of SST+/CR+ and CR+ cells at 100 DIV in the three experimental conditions (n=3) One way ANOVA and post-hoc Tukey test. IN Only vs. IN + hGPCs p>0,9999 ns; IN Only vs. IN + mAST p>0,9999 ns; IN + hGPC vs. IN + mAST p= 0,8902 ns. **I)** Heatmap showing relative gene expression levels of *SST* and *TH* at different time points across a differentiation protocol with H9 cells. **J)** Z-stacks from confocal imaging, showing the distribution of SST+ cells throughout the whole spheroid at 50 DIV. Scale bar = 100 µm. **K)** Immunostaining showing the co-localization of SST with TH at 50 DIV. **L)** Quantification of SST+ and SST+/TH+ cells at 50 DIV (n=1). DIV=days *in vitro*; hIN=human interneurons; hGPC=human glial progenitor cells; mAST=mouse astrocytes.

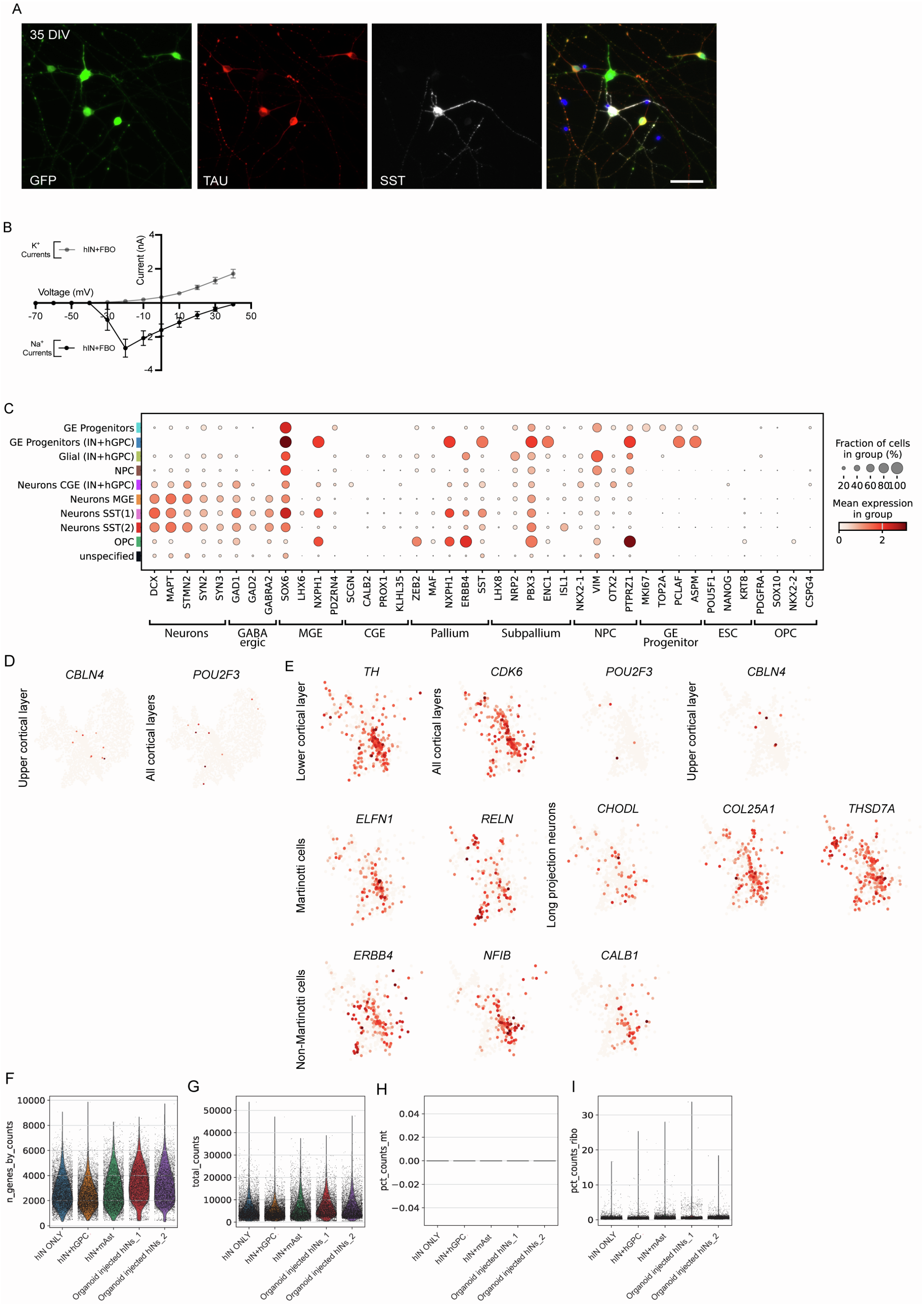

Figure S4. Characterization of organoid injected hINs. Related to Figure 4. A) Immunostaining showing hESC-derived MGE-like progenitors prior to injection expressing GFP, neural marker TAU and SST at 35 DIV. **B)** Inward Na+ and outward K+ current plotted against voltage injection steps for GFP+ injected cells in human FB organoids. **C)** Dot plot showing the expression of genes representative for

each cluster of Figure 4I. Dot plot is similar to the format presented in Bershteyn et al., 2024. **D)** UMAP plots showing the expression of *CBLN4* and *POU2F3* on SST population derived from cells from spheroids across conditions at 100 DIV and injected GFP+ cells at 100 DPI **E)** UMAP plots showing expression of cortical layer-specific markers, Martinotti cell-specific markers, long projection neuron-specific markers and non-Martinotti cell-specific markers on SST population derived from cells from injected GFP+ cells at 100 DPI. **F)** Number of genes by counts per sequencing sample. **G)** Total counts per sequencing sample. **H)** Mitochondrial counts per sequencing sample. **I)** Ribosomal counts per sequencing sample. DIV=days *in vitro*; DPI=days post injection; hIN=human interneurons; hGPC=human glial progenitor cells; mAST=mouse astrocytes; FB=forebrain.

Table S1. See separate excel file. Statistical analysis of gene expression measured by qPCR. Values reported are p-values between the given groups and time points from 4 independent experiments (replicates).

Table S2. List of primary antibodies used.

Primary Antibodies			
**Antibody**	**Concentration**	**Company**	**Ref #**
**Chicken anti-GFAP**	1:2000	Merck Millipore	AB5541
**Goat anti-PDGFRα**	1:300	R&D Systems	AF-307-NA
**Chicken anti-MAP2**	1:2000	Abcam	ab5392
**Mouse anti-SST**	1:200	Santa Cruz Biotechnology	sc55565
**Rabbit anti-CALB-2**	1:200	Abcam	ab702
**Mouse anti-MAP2**	1:300	Merck Millipore	MAB 3418
**Rabbit anti-TTF-1/Nkx2.1**	1:500	Novus Biologicals	8G7G3
**Rabbit anti-TH**	1:1000	Merck Millipore	AB152
**Mouse anti TUJ-1/BIII**	1:1000	Biologend	801211
**Rabbit anti-GABA**	1:2000	Sigma-Aldrich	A2052

Table S3. List of secondary antibodies used.

Secondary Antibodies			
**Secondary**	**Concentration**	**Company**	**Ref #**
**Alexa Fluor 488 Donkey anti-Mouse**	1:200	Jackson ImmunoResearch	715-545-150
**Alexa Fluor 488 Donkey anti-Rabbit**	1:200	Jackson ImmunoResearch	711-545-152
**Alexa Fluor 568 Donkey anti-Rabbit**	1:500	Invitrogen	A10042
**Alexa Fluor 568 Donkey anti-Mouse**	1:500	Invitrogen	A10037
**Alexa Fluor 647 Donkey anti-Chicken**	1:200	Jackson ImmunoResearch	703-605-155
**Cy2 Donkey anti-Mouse**	1:200	Jackson ImmunoResearch	705-225-147

Table S4. List of primers used.

List of Primers		
**Gene**	Gene name	Primers (forward/reverse)
ACTB	Beta-actin	CCTTGCACATGCCGGAG<br>CCTTGCACATGCCGGAG
ASCL1	Achaete-Scute Family BHLH transcription factor 1	CTAAAGATGCAGGTTGTGCG<br>GGAGCTTCTCGACTTCACCA
ARX	Aristeless Related Homeobox	CCTGAGCACTTTCCTCGGAGCG<br>TGGAAAAGAGCCTGCCGAATGCC
CALB1	Calbindin 1	TGGCTCCATTTCGACGCTGACG<br>ATCCAGCCTTCTTTCGCGCCTG
CALB2	Calbindin 2	TGGAGGCTTGGCGGAAGTACGA<br>CCGGTTCGCCTTCTTCAGCAGG
CBLN4	Crebellin 4 precursor	CCGAGATGAGCAACAAGACG<br>CCCCCGCAAAGGCAGATATT
CCK	Cholecystokinin	AGGGTATCGCAGAGAACGGA<br>CTTATCCTGTGGCTGGGGTC
CDK6	Cyclin depedent kinase 6	TGCACAGTGTCACGAACAGA<br>ACTATAGATGCGGGCAAGGC
CHODL	Chondrolectin	ACAGATGAACCTTCCTGCGG<br>TCTGATGGGTGTCTCCTGGT
DLX2	Distal-Less Homeobox 2	ACCAGACCTCGGGATCCGCC<br>CTGCGGGGTCTGAGTGGGGT
DLX5	Distal-Less Homeobox 5	GCCTCCGGGACACTCTATTA<br>CAGTTTTCCGAACTTCCCCAT
FOXG1	Forkhead Box G1	CTGTCTGGGGGACTGTCTGAT<br>GTCTGGTCCCAGGGATGTT
GAD2	Glutamate decarboxylase 2	ATCCTCACGACTCAGCTCCC<br>GAGCTTTAAAAGAGACCGGGACT
GAPDH	Glyceraldehyde-3-Phospahte Dehydrogenase	TTGAGGTCAATGAAGGGGTC<br>GAAGGTGAAGGTCGGAGTCA
GFAP	Glial Fibrillary Acidic Protein	TCATCGCTCAGGAGGTCCTT<br>CTGTTGCCAGAGA TGGAGGTT
KCNC1	Potassium Voltage-Gated Channel Subfamily C Member 1	GCTGTTCGAGGACCCCTACT<br>GTTGAAGCGCTCGTGAGTCT
LXH6	Lim Homeobox 6	CACTTCCGCATCTGCCCGT<br>CGCAGCTTGGACACTGGATCT
MAF	MAF BZIP Transcription Factor	CACTCGCAAGTTGGAGCCAT<br>ACTGGTAAGTACACGATGCTGG
MAFB	MAF BZIP Transcription Factor B	CGCTGGCCATGGAGTATGTC<br>GTGTCTTCTGTTCGGTCGGG
MAP2	Microtubule Associated Protein 2	CCGTGTGGACCATGGGGCTG<br>GTCGTCGGGGTGATGCCACG
MEF2C	Myocyte Enhancer Factor 2C	TCTCTCCCTGCCTTCTACTCA<br>GGAGTGGAATTCGTTCCGGT
MYH8	Myosin Heavy Chain 8	AGAAGCCACTGGGCATCTTC<br>TACGAAGTGAGGGTGTGTGC
NKX2.1	NK2 Homeobox 1	AGGGCGGGGCACAGATTGGA<br>GCTGGCAGAGTGTGCCCAGA
NPY	Neuropeptide Y	TGTTCCCAGAACTCGGCTTG<br>TGCATTGGTAGGATGGGTGG
PDGFRA	Platelet-Derived Growth Factor alpha	CCTTGGTGGCACCCCTTAC<br>TCCGGTACCCACTCTTGATCTT
POU2F3	POU Class 2 Homeobox 3	ATGCGCGGCTCCTTTAACCGG<br>TTAGACGCTGCGGTCGCCATG
PVALB	Parvalbumin	TGCAGGATGTCGATGACAGA<br>TTTCTTCAGGCCGACCATTT

SLC32A1	Solute Carrier Family 31 Member 1	AGATGATGAGAAACAACCCCAG<br>CACGACAAGCCCAAAATCAC
SST	Somatostatin	CAAGCCGCTTTAGGAGCGAG<br>AGGCGGCAGGACAGCATCT
SYN1	Synapsin 1	CCCGTGGTTGTGAAGATGGGGC<br>TGCCACGACACTTGCGATGTCC
TACSTD2	Tumor Associated Calcium Signal Transducer 2	CCACCAACAAGATGACCGTG<br>CAGCAGACACTTGGAGGTCA
TBR1	T-Box Brain Transcription Factor 1	TCGTCCCCGCTCAAGAGCGA<br>CCTTGGCGCAGTTCTTCTCGCA
TH	Tyrosine Hydroxylase	CGGGCTTCTCGGACCAGGTGTA<br>CTCCTCGGCGGTGTACTCCACA
VIP	Vasoactive Intestinal Peptide	TCTCACAGACTTCGGCATGG<br>TCATTTGCTCCCTCAAAGGGT

SUPPLEMENTAL EXPERIMENTAL PROCEDURES

Cell cultures and generation of interneuron spheroids

hESCs were differentiated into interneurons according to Fig. 1. In brief, cells were plated in iPS Brew XF medium with ROCK inhibitor in ultra-low attachment round-bottom 96-well plates (Corning, New York, NY, USA) to form embryoid bodies. After one day, the media was replaced with Neurobasal-A medium supplemented with B27 Vitamin A and Non-essential amino acids (all from Thermo Fisher, Waltham, MA, USA). The following patterning factors were added to the media: Dickkopf WNT Signaling Pathway Inhibitor 1 (DKK1; 1 μg/mL, R&D Systems, MN, USA), SB-431542 (10 μM, Axon, Groningen, Netherlands), Noggin (0.5 μg/mL, R&D Systems, Minneapolis, MN, USA) and purmorphamine (1μM; Merck, Damstadt, Germany). On day 9, embryoid bodies were plated into polyornithine/laminin-coated 6-well plates and allowed to attach. All patterning factors except purmorphamine were removed on day 14. On day 25, cells were detached and replated as a monolayer. Notch inhibitor compound-E was added on day 27 to push the cells out of the cell cycle and promote further maturation. At 35 days after the start of the differentiation, cells were detached, and 90,000 or 100,000 cells were seeded in each well of a 96-well ultra-low attachment plate to self-aggregate into spheroids. At this point, glia co-culture was initiated by adding either 10,000 mouse astrocytes (mAST; ScienCell, CA, USA) or 10,000 human glial progenitor cells (hGPCs). Interneuron spheroids were then kept in Neurobasal-A media supplemented with B27 Vitamin A, Non-essential amino acids with BDNF (25 ng/ml).

Generation of hGPC

On day 0 the hESCs were cultured in iPS-Brew XF medium (StemMACS, Miltenyi, Bergisch Gladbach, Germany) in ultra-low attachment flasks for embryoid body (EB) formation. By day 5, the medium was switched to neural induction medium (NIM) and on day 9, the EBs were transferred to polyornithine/laminin coated plates. From day 11, NIM with RA (0.1 μM, Sigma-Aldrich) was used, and on day 16, the medium was switched to NIM/B27 medium containing RA and purmorphamine (1 μM, Millipore). Between days 26-29, EBs were detached, seeded as clusters in ultra-low attachment plates and kept in NIM/B27 medium with purmorphamine and bFGF (10 ng/ml). On day 37, the medium was changed to glial medium (GM) containing gliogenic factors; PDGF-AA protein (10 ng/ml, R&D Systems), IGF-I (10 ng/ml, R&D Systems) and NT-3 Protein (10 ng/ml, R&D Systems), and supplemented with purmorphamine, which was removed on day 55. The cells were then kept in GM until the end of differentiation. On day 70, the floating clusters were manually cut under a dissection microscope and attached onto PO/Lam-coated plates. This process was repeated every 30 days until the end of the differentiation. Before co-culture, hGPCs were analyzed by FACS and consisted of 44% CD140a+ cells (marking PDGFRα+ cells), 0.5% CD44+ cells (marking astrocyte-biased cells) and 0.3% bipotent CD140a+/CD44+ cells. The hGPCs were added to the co-culture with interneurons on day 186 of their differentiation.

Immunofluorescent staining and clearing procedure

Before staining, cells were rinsed 3 times with phosphate-buffered saline (PBS) and afterwards incubated with blocking buffer (0.1% Triton X-100 [Sigma-Aldrich, St. Louis, MO, USA], 5% donkey serum in PBS) for 1 hour. After blocking, cells were incubated with primary antibodies (Table S2) overnight at 4 °C. Subsequently, cells were washed 3 times with PBS and incubated with secondary antibodies (Table S3) for 1h at RT. Spheroids were stained both as cryosections and as whole spheroids. The spheroids and organoids were incubated overnight at RT in a blocking solution containing 5% donkey serum and 0.5% Triton X-100 in KPBS. The spheroids were then incubated for 3 days at RT with the corresponding primary antibodies (Table S2) diluted in the blocking solution. Following this, spheroids and organoids were rinsed three times with KPBS and incubated for 3 days at RT with the secondary antibodies (Table S3) diluted in a blocking solution. The spheroids and organoids were then rinsed three additional times with KPBS. Spheroids were preserved at 4°C until clearing was performed. The stained spheroids were dehydrated through a progressive series of methanol solutions (20%, 40%, 60%, 80% and 100%), allowing them to sit in each solution for 10 minutes. After dehydration, spheroids were incubated in a mixture composed of methanol and dichloromethane (DCM), followed by two 10-minute washes with DCM. The clearing was completed by washing the spheroids with ethyl cinnamate. Finally, spheroids and organoids were carefully transferred into 96-well thin bottom plates for imaging. Quantification of SST, TH and CR expressing cells was performed manually in ImageJ (NIH, Bethesda, MD, USA) by counting the positive cells throughout the confocal z-stacks, acquired with a 2.3μm interval between optical planes. The total number of stacks was on average 120-130 depending on the size of the spheroid. Data were normalized per stack for analysis.

Generation of FB organoids and interneuron injection

Briefly, hESC cultures at 75-90% confluency were dissociated, counted and seeded at a density of 8,000 cells/well in an ultra-low attachment 96-well plate (Costar, round bottom, REF 7007) with a final volume of 25 μl/well. After 3 days, the medium was replaced with a neuronal medium as previously described (Lancaster *et al.*, 2017; Sozzi *et al.*, 2022). On day 8, each organoid was embedded in 30 μl of Matrigel (Corning, 354234) and incubated

at 37°C for 25 min for Matrigel polymerization. The organoids were then transferred to an ultra-low attachment plate (Corning, flat bottom, REF 3471) with media as reported before (Lancaster *et al.*, 2017; Sozzi *et al.*, 2022). At 35 DIV, human FB organoids were injected with GFP expressing MGE-like progenitors of the same developmental age under a dissection microscope using a Hamilton syringe, as previously reported (Reumann *et al.*, 2023). The injected FB organoids were transferred to 24-well ultra-low attachment plates at 37°C with 5% CO_2. The media was refreshed every 2-3 days, with 80% of the volume replaced each time.

Cryosectioning

Spheroids were fixed with 4% paraformaldehyde (PFA) and placed in a 30% sucrose solution for cryoprotection overnight. The following day, the spheroids were transferred to a 1:1 mixture of OCT and sucrose for 2 hours. The spheroids were then embedded in OCT and placed on dry ice. Once embedded, the spheroids were sectioned to a thickness of 14 or 20 µm using a cryostat (Cryostar NX70, Epredia, Portsmouth, New Hampshire) and stored at -20 °C until analysis.

RNA Extraction, cDNA synthesis and Quantitative Real-Time Polymerase Chain Reaction (RT-qPCR)

Total RNA from the samples was extracted with the RNeasy Micro Kit (Qiagen, Hilden, Germany), following the manufacturer's instructions. For reverse transcription, the Maxima First Strand cDNA Synthesis Kit (Thermo Fisher, Waltham, MA, USA) was used, and 500 ng RNA from each sample were reverse transcribed according to the manufacturer's protocol. The Bravo Automated Liquid Handling Platform (Agilent, Santa Clara, CA, USA) was used to prepare the PCR mix in each well of a 384-well plate: cDNA (1 µl), LightCycler 480 SYBR Green I Master (5 µl, Roche, Basel, Switzerland), and relevant primers (4 µl, Table S4). This mix was subsequently analysed by RT-qPCR on a LightCycler 480 II instrument (Roche, Basel, Switzerland) using a two-step protocol (95 °C, 30 s denaturation and 60 °C for 1 min annealing/elongation) for 40 cycles. The relative gene expression was calculated from technical triplicates, using the ΔΔCT method, comparing expression to undifferentiated cells (hESCs) and normalizing against two housekeeping genes (*ACTB* and *GAPDH*). A heatmap was generated using R software (version 4.3.3). Gene expression data was scaled on a logarithmic scale to ensure proper data representation. For heatmap visualization, the heatmap package (version 1.0.12) was used. The average from four individual samples from each time point and condition is visualized in the heatmaps.

Nuclei isolation from spheroids, isolation of GFP+ cells from the organoid and FACS-based sorting

Spheroids were gradually thawed on ice and dissociated in ice-cold lysis buffer (0.32 M sucrose, 5 mM $CaCl_2$, 3 mM MgAc, 0.1 mM Na_2EDTA, 10 mM Tris-HCl pH 8.0, 1 mM DTT, 0.1% Triton X, EDTA-free proteinase inhibitor [Roche, Basel Switzerland] and RNAse inhibitors [Ambion ™ and SUPERase In™, Invitrogen, Carlsbad, CA, USA]). Lysates were centrifuged at 11,000 x g for 30 min at 4°C. The pellets were resuspended in a sorting buffer consisting of 0.1% BSA Fraction V, PBS -/-, RNAse inhibitors (Ambion ™ and SUPERase In™, Invitrogen, Carlsbad, CA, USA) and Draq7™ (BD Biosciences no. 564904, Eysins, Switzerland). The nuclei were filtered through a 70 µm filter into BSA-coated DNA LoBind tubes (Eppendorf, Hamburg, Germany) for sorting. Nuclei sorting was performed with a FACSAria cell sorter with a 100 µm nozzle and FACS Diva software (BD Biosciences, Eysins, Switzerland) at a low flow rate to isolate single nuclei. 12,000 nuclei were collected from each sample and directly processed for cDNA library generation. During FACS, GFP+ nuclei were identified using an FB organoid that was not injected as a negative control.

Electrophysiological recordings

Cells with a clean surface and clear access paths were selected for recordings. The resting membrane potential was noted immediately after opening the cell membrane in current-clamp mode. Thereafter, cells were kept at a membrane potential of -65 mV to -70 mV. For evoked action potentials, cells were injected with 500 ms currents from -20 pA to + 35 pA with 5 pA increments, and ramp injection of 100 pA. Measurements of inward sodium and delayed rectifying potassium currents were done in voltage-clamp mode while holding the cell at -70 mV and applying voltage-depolarizing steps for 100 ms from -70 mV to 40 mV at 10 mV increments. Spontaneous activity was recorded in voltage-clamp mode at −70 mV. The baseline of the traces was adjusted manually in Clampfit 10.3 and filtered with a Gaussian low-pass filter (cutoff 110 Hz; Molecular Devices, San Jose, CA, USA). Picrotoxin (PTX) was added to the external solution at a final concentration of 100 µM. Action potential properties were measured from the first evoked spike exceeding 10 mV in height following rheobase current injection steps. Data were analyzed using Clampfit 10.3 (Molecular Devices, San Jose, CA, USA) and Igor Pro 8.04 (Wavemetrics, Portland, OA, USA), combined with the NeuroMatic package (Rothman and Silver, 2018).

snRNA-seq data analysis

Spheroid and organoid datasets were concatenated separately, and highly variable genes were identified using 'sc.pp.highly_variable_genes'. The data were then rescaled, followed by Principal Component Analysis (PCA), and batch effects were corrected with BBKNN (batch-balanced K Nearest Neighbours). For the spheroid data, the 10 nearest neighbours were computed using 50 principal components, and clustering was performed using the

Leiden algorithm. To integrate data from spheroids and injected cells, common genes were identified, and integration was performed with 'scanpy.tl.ingest'. Label transfer was used to assign cluster annotations from the spheroid dataset to the injected cell dataset based on previously determined clusters annotated using marker genes. The SST datasets were extracted from the neuron clusters of spheroid and injected cell datasets, respectively, based on normalized SST expression greater than 1. These SST subsets were then integrated following the same procedure as the full datasets. Nuclei were visualized using UMAP (Uniform Manifold Approximation and Projection). Dot plots were generated with Scanpy, and bar plots were created with Matplotlib (v3.7.2).

- Rothman, J.S., and Silver, R.A. (2018). NeuroMatic: An Integrated Open-Source Software Toolkit for Acquisition, Analysis and Simulation of Electrophysiological Data. Front Neuroinform *12*, 14. 10.3389/fninf.2018.00014.
- Lancaster, M.A., Corsini, N.S., Wolfinger, S., Gustafson, E.H., Phillips, A.W., Burkard, T.R., Otani, T., Livesey, F.J., and Knoblich, J.A. (2017). Guided self-organization and cortical plate formation in human brain organoids. Nat Biotechnol *35*, 659-666. 10.1038/nbt.3906.
- Sozzi, E., Kajtez, J., Bruzelius, A., Wesseler, M.F., Nilsson, F., Birtele, M., Larsen, N.B., Ottosson, D.R., Storm, P., Parmar, M., and Fiorenzano, A. (2022). Silk scaffolding drives self-assembly of functional and mature human brain organoids. Front Cell Dev Biol *10*, 1023279. 10.3389/fcell.2022.1023279.
- Reumann, D., Krauditsch, C., Novatchkova, M., Sozzi, E., Wong, S.N., Zabolocki, M., Priouret, M., Doleschall, B., Ritzau-Reid, K.I., Piber, M., et al. (2023). In vitro modeling of the human dopaminergic system using spatially arranged ventral midbrain–striatum–cortex assembloids. Nature Methods *20*, 2034-2047. 10.1038/s41592-023-02080-x.
